# Honey as a Natural Nutraceutical: Its Combinational Therapeutic Strategies Applicable to Blood Infections—Septicemia, HIV, SARS-CoV-2, Malaria

**DOI:** 10.3390/ph16081154

**Published:** 2023-08-14

**Authors:** Caoimhin Mackin, Divakar Dahiya, Poonam Singh Nigam

**Affiliations:** 1 Biomedical Sciences Research Institute, Ulster University, Coleraine BT52 1SA, UK; 2 Wexham Park Hospital, Wexham Street, Slough SL2 4HL, UK

**Keywords:** nutraceutical, honey, blood, therapy, sepsis, HIV, SARS-CoV-2, COVID-19, malaria

## Abstract

Honey is a natural substance that has existed alongside humanity since the time of antiquity, acting then as a source of nutrition, as well as a source of medicinal aid for people. Ancient civilizations from multiple nations of the world, from ancient China to ancient Greece and Egypt, utilized the supposed healing properties of honey to treat lacerations and wounds, as well as for internal pathologies such as intestinal disease. At present, honey has entered the modern scientific research program in search of novel antibiotics. In recent research, honey has demonstrated its potential use for static and/or cidal effects on microbial strains which are becoming resistant to chemical antibiotics. Additionally, the use of honey as an agent of treatment for more severe infections, namely blood infections pertaining to septicemia, HIV, and SARS-CoV-2, as well as parasitic infections such as malaria, have also been investigated in recent years. In this article, the literature has been reviewed on some of the therapeutic properties of natural nutraceutical honey, where it has been observed to act as a potential ameliorating agent; reducing the severity of such conditions that may amplify a disease, as well as reducing the progression of the disease and its symptoms.

## 1. Introduction

Antibiotic and antimicrobial resistance is a ubiquitous and ever-evolving threat to the health of the public. It is a silent and multi-faceted plague that has, according to current statistics and data, killed 1.27 million people in antibiotic-resistance-related deaths worldwide, with nearly 5 million direct deaths within 2019 [1]. In the US, 2.8 million antibiotic-resistant infections occur yearly, and of those 2.8 million 35,000 people die annually as a direct result of those infections [2]. In the UK, antibiotic-resistant infections increased by 2.2% in 2021–illustrated as an increase from 52,842 infections in 2020 to 53,985 infections in 2021 [3]. Combined with the statistics of western European deaths, 51,000 people died within 2019 as a direct consequence of an antibiotic infection [4].

The international collaborative efforts that were demonstrated so readily during the COVID-19 pandemic are proof that the medical sectors, the health departments, and the world are prepared to devote all resources and efforts towards accomplishing a unified goal. If cooperation and collaboration on such a scale were achieved again, with investigation into the many natural and synthetic materials that may produce exploitable antibiotic effects, the millions of deaths which occur annually would be avoided, if not significantly reduced, within a narrow window of time.

Of the many materials which are being, or have been, routinely evaluated and assessed, one of the most promising is honey. Honey is a naturally occurring sweet substance that is produced from flower nectar processed through the upper digestive tract of the honeybee. The exact composition of honey varies between the geographical region of production, given the flowers and plants which have been gathered from, as well as between the species of bee which have harvested the nectar, though the general composition of honey is 80% sugars, 20% water, and a varying percentage of other constituents such as vitamins, minerals, flavonoids, proteins, amino acids, peptides, enzymes, and phenolic acids. The sugars in honey are represented by monosaccharides, glucose and fructose, followed by disaccharides, sucrose, maltose, turanose, isomaltose, maltulose, trehalose, nigerose, kojibiose and trisaccharides maltotriose, and melezitose. Disaccharides and trisaccharides like sucrose and maltotriose are hydrolyzed enzymatically to monosaccharides. Sucrose consists of one molecule of fructose linked with glucose through α-1,4 binding [5].

Honey has been used intrinsically throughout human history as a source of inspiration, sustenance, and as a source of reliable medicinal application–with reference to the material being recovered from the artefacts originating from the time of the ancient Greeks, Romans, Chinese, and Egyptians, who all invoked the honey’s property to treat intestinal conditions, contusions, and superficial wounds and lacerations [6]. The applications, in which honey has been and can be used, vary between beliefs within alternative medicine to clinical-based scenarios depending on chemical composition and geographical location [7]. One of the most documented, and widely accepted, uses of honey as a form of medicinal material is that of its properties within and pertaining to wound healing, or wound management [8]. The Ebers Papyrus, originating from 1500BCE, describes a recipe for a topical medicine made from vegetable fibers, animal fat, and, most importantly, honey [9]. The vegetable fibers would function as an absorbing material for the wound dressing; the honey functioned as the wound dressing itself, and provided aid in its antibiotic and healing properties, and the animal fat functioned as a natural barrier between the dressing/treatment and the outside environment.

**Figure 1:**
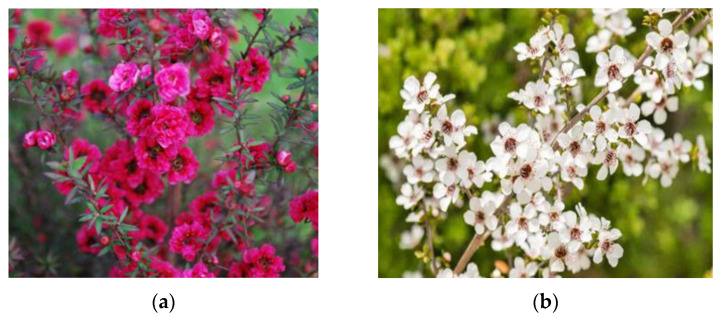
(**a**,**b**): Two varieties of Flowers of Leptospermum scoparium shrub. Source of pictures: (**a**) https://thessfyta.gr/en/ornamental-%CE%B2ushes/73-leptospermum-scoparium.html. (**b**) https://plantcaretoday.com/leptospermum-scoparium.html. Pictures accessed on 14 June 2023.

Within recent studies and publications, a revitalization has taken place with the reintroduction and reassessment of honey as a potential agent of antibiotic delivery. The most accepted of the potential antibiotic kinds of honey is manuka honey, a rich-golden, mono-floral honey. It is produced only by a specific species of honeybee, *Apis mellifera*, with only the nectar from a plant, *Leptospermum scoparium* [10], that has reinforced itself within the spirit of the time of the modern scientific community, due to the documented antioxidant, anticancer, and antimicrobial bioactivity of the honey’s constituents [11]. *Leptospermum scoparium* is a species of flowering shrub (Figure 1) in the myrtle family Myrtaceae, native to New Zealand; its nectar produces manuka honey.

## 2. Bioactivities of Honey

The antioxidant property of manuka honey is a result of the high level of phenolic compounds within the substance, with which these compounds possess a ready capacity to capture and reduce free radicals. Of the phenolic content of honey, a study conducted by Lawag, et al. on four separate species-derived honeys from western Australia—*Calothamnus* spp., *Agonis flexuosa*, *Corymbia calophylla*, and *Eucalyptus marginata* honeys–found a variety of phenolic compounds both shared and unique amongst the different types of honey [12]. Of these four western Australian honeys, some of the phenolic compounds, which were identified and quantified, were as follows: t-Cinnamic acid, Eudesmic acid, m-Coumaric acid, Kojic acid, Lumichrome, Gallic acid, Taxifolin, Syringic acid, and Protocatechuic acid [12]. Similarly to the study by Lawag, et al., Farkas, et al. conducted a study to determine the phenolic content within four Hungarian varieties of honey—*Robinia pseudoacacia*, *Asclepias syriaca*, *Tilia* spp., and *Solidago gigantea*–that have, not by their record, been analyzed or evaluated in studies past attempting to categorize the phenolic make-up of honeys as a form of identifier or fingerprint [13]. Of these four Hungarian honeys, some of the phenolic compounds are as follows: Syringic acid, Taxifolin, Vanillic acid, p-Coumaric acid, Gentistic acid, Chrysin, Galagin, Caffeic acid, and Hesperetin [13]. As illustrated by the completed works of Lawag, et al. and Farkas, et al., honey, regardless of the geographical origin, is diversely rich in antioxidant, bioactive phenolic compounds—chiefly, both works of honey evaluation revealed matching phenolic constituents, despite being both miles separated and different in botanical origin; namely, Syringic Acid and Taxifolin [12,13]. 

Further antioxidant bioactivity, as well as having similar potential to act as a fingerprinting model, has been recorded to stem from the presence of organic acids within honey. Organic acids are not major constituents of honey, approximately accounting for <0.5% of the constituent volume of the material [14]. The biological activity of these organic acids extends outside of the potentiality of nutraceutical applications within humans; in the wild, the generation of nonaromatic organic acids within honey prevents the development of bee-specific disease and death of the hive, as well as organic acids, which also act as a marker or indicator of fermentation to allow for determination of stability of honey within commercial environments, and organic acids, as stated previously, can be utilized much like phenolic compounds to act as a fingerprint model for the identification of botanical origins of honey samples [14]. There are five main nonaromatic organic acids that are known to be present within honey across different geographical locations: Gluconic acid, Tartaric acid, Malic acid, Citric acid, and Succinic acid [14]. Of these five, Gluconic acid is the organic acid which is found in the greatest abundance within various honey samples from different geographical locations—accounting for 64.6–99.8% of the total organic acid volume within a sample [15]. 

The antioxidant activity of Gluconic acid, and other organic acids such as Citric and Malic acid, derives from their ability to work synergistically with other antioxidant compounds, such as phenolic compounds, by facilitating the chelation of heavy metal cations via their carboxyl group structures (−COOH) and furthering their combined antioxidant potential [14,16,17,18].

The antiproliferative effect of manuka honey on cancer cells has been demonstrated within in vitro simulations with colon (HCT-116), lung (A549), and breast (MCF-7) cancer cell lines on a dose-dependent basis [19], and the antimicrobial property of manuka honey, particularly the non-peroxide-based antimicrobial ability, has been credited to its distinct constituent methylglyoxal [11].

Methylglyoxal, abbreviated as MGO and denoted in molecular structure as CH3C(O)CHO or C3H4O2, is a 1,2-dicarbonyl compound, formed from dihydroxyacetone [20,21], that has demonstrated a significant bacteriostatic effect on resistant strains of bacterial species, such as multi-drug resistant *Pseudomonas aeruginosa*, by bypassing and remaining unrecognized by bacterial efflux mechanisms [22]. MGO demonstrates no discrimination in its ability to produce an antibiotic effect between Gram-negative and Gram-positive bacteria–it was concluded that, though Gram-negative species such as *Escherichia coli* may be more prone to destruction by way of the osmotic effects of manuka honey, MGO demonstrates synergy with other antibacterial compounds in manuka honey to inhibit *Staphylococcus aureus*, *Escherichia coli*, and *Pseudomonas aeruginosa* [23]. 

The bioactive properties of honey, and the effects it can impose upon other living organisms such as microorganisms, is not a novel discovery; technological and evaluative advances have allowed for precise and accurate analysis and assessment of the entire profile and scope of these bioactive properties—allowing, also, for the determination of limits, both physical and biochemical, about these same properties. Of manuka honey’s known inclination to act as a reducing agent with an antioxidant capacity, evaluations have since been conducted to further investigate this characteristic. An evaluation report published by Yusof et al. sought to produce effective methods employed to determine the total antioxidant capacity of materials—specifically with ingestible materials including honey [24]. Specifically, researchers evaluated the iron (III) reducing the antioxidant capacity of five types of manuka honey (5 + UMF, 10 + UMF, 15 + UMF, 18 + UMF, and a nonrated UMF), using several total antioxidant capacity assays; one of these involved tracking of chemical kinetics with the compound ABTS. Researchers concluded that, through their assay evaluations, a regular variation of antioxidant capacity of all manuka honey samples was observed between all assay formats. This study confirmed bioactivity and antioxidant capacity in all honey samples; however, this leads to the necessity of further investigation of manuka honey’s antioxidant bioactivity and capacity based on conditions of the assay [24].

Further evaluations have been made in hopes of determining a link between the phenolic profile of some kinds of honey, and their antibacterial and antioxidant capacity. Chau et al. studied the antioxidant capacity and antibacterial capacity of extract samples prepared from manuka honey in comparison to the identical evaluations conducted on an unfractionated form of the same manuka honey [25]. Results concluded that the manuka honey extract, gathered through ethyl acetate extraction, possessed a 30× greater total phenolic content compared to that of the unfractionated manuka honey sample; the total antioxidant capacity of the manuka honey extract demonstrated a nearly 100× greater capacity, as well [25]. 

However, in terms of the antibacterial property of these two materials, the unfractionated manuka honey sample demonstrated zones of inhibition 2× larger than that of the manuka honey extract when evaluated through a disc diffusion assay; a 96-well microtiter assay agreed with concordant results that the unfractionated sample of manuka honey possessed a greater antibacterial capacity than the manuka honey extract [25]. Though the manuka honey extract was shown to have a richer polyphenol profile and greater antioxidant capacities, the unfractionated sample demonstrated greater antibacterial properties. An extrapolation could be made from this evaluation that the phenolic profile and bioactive properties of honey may be mutually exclusive characteristics and that a concise and definitive link does not exist between the two; although, this itself creates the necessity for further study and research to strengthen such a conclusion between total phenolic profile, total antioxidant capacity, and antibacterial properties [25,26]. 

A summary of the bioactive properties of honey mentioned within this section, and further novel applications, is presented in Table 1. 

## 3. Blood Infection 

### 3.1. Septicemia/Sepsis

Sepsis is an extremely serious medical emergency that occurs when the body is already suffering from an infection and is most likely to originate from infections of the lungs, urinary tract, skin, and gastrointestinal tract [36]. Without receiving necessary treatment for any current infections, sepsis may develop and rapidly progress and cause systemic organ failure, tissue damage, and death. Sepsis is a systemic illness caused by an invasion of a pathogenic microbe into a normally sterile area of the body—resulting in a detrimental and damaging response of the host innate immune system towards said infection. The condition begins when the host’s immune response towards a pathogen becomes amplified dramatically and dysregulated [37]. At this stage, initial symptoms may manifest, such as fever, confusion, transient hypotension, restricted urine output, as well as thrombocytopenia—a platelet deficiency within the blood, characterized as a reduced ability for blood to clot [37]. If left untreated, symptoms will progress to renal and pulmonary failure, as well as a severe coagulation complication—principally Disseminated Intravascular Coagulation (DIC), a generally rare condition that causes abnormal coagulation throughout the body’s blood vessel system [38]. 

Sepsis begins with the introduction of a Pathogen Associated Molecular Pattern (PAMP) to the host’s immune system when a pathogen first colonizes a host, where macrophages of the innate immune system can detect the presence of these PAMPs and in turn become stimulated. From these activated macrophages, pro-inflammatory cytokines—namely IL-10 and IL-6–Nitric Oxide—and Prostaglandins are secreted to induce localized inflammation to halt the growth and reproduction of the pathogen. Septicemia, also referred to as toxemia or blood poisoning, is the condition in which bacteria infect a person’s bloodstream and begin to reproduce, colonize, and release their associated toxins into their host’s system [39]. As stated, septicemia is when a pathogenic organism is able to enter and infect a person’s bloodstream, and is able to progress to sepsis or septic shock, while sepsis is an aggressive and heightened response to an infection [40]—while used interchangeably, especially within a casual context, the two conditions and processes are not the same. 

#### 3.1.1. Combinational Therapy for Sepsis Using Nutraceutical Honey 

Manuka honey was reported to possess several therapeutic properties, based on its antioxidant power [41], phenol content [42], and methyl glyoxal equivalents determining its manuka power [43]. With its recognized properties, the potential effect of Chrysin (5,7-dihydroxyflavone) found in honey was evaluated on LPS-induced sepsis within six groups of male rats (*n* = 10) by Koc et al. [44]. Chrysin, molecular formula C_15_H_10_O_4_, is a known plant flavonoid, widely sourced from propolis, mushrooms, honey, and various other species of plants, and is known for its profound bioactivity within antioxidant, antispasmodic, anti-inflammatory, and anxiolytic capacities [44]. It is understood that the anti-inflammatory activity of Chrysin is due to its ability to inhibit COX-2 expression, as well as its ability to interact with IL-6 signaling. Koc et al. subjected rat model Group 1 to 1 mL of corn oil as a control, Group 2 to only intraperitoneal injection of LPS, derived from *Escherichia coli* O111: B4 at 100 µg/kg by weight; Group 3 and 4 to intraperitoneal injection of LPS at 100 µg/kg by weight with Chrysin solutions at 50 mg/kg and 100 mg/kg, respectively, and Groups 5 and 6 to oral doses of Chrysin in corn only at 50 mg/kg and 100 mg/kg, respectively. 

Blood samples and organ tissues after 10 days of supplementation with LPS and Chrysin were extracted and homogenized, then subjected to histopathological and serum analysis [44]. Through comparisons of groups post-analysis, it was concluded that the groups given the Chrysin solutions, as well as the injections of LPS, showed significantly reduced levels of IL-1β, IL-10, Tumor Necrosis Factor-α, Aspartate Transaminase, Alanine Aminotransferase, IL-6, and Malondialdehyde. Chrysin-supplemented groups also showed to increase the levels of Superoxide Dismutase, Catalase, and Glutathione Peroxidase—intracellular enzymes responsible for the protection of cells and tissue against radical oxidative stress, as well as maintenance of cell signaling [45,46,47]. The study concluded that Chrysin had potential in reducing the oxidative stress markers, as well as cytokine levels, amplified within the process of sepsis—noting the significant reduction of sepsis-associated acute tissue injury after continuous supplementation of Chrysin, suggesting potential applications as a natural pharmaceutical agent for other inflammatory diseases [44].

The potential anti-inflammatory and antibacterial effect of three different types of Greek honey, Arbutus, Chestnut, and Fir, with control manuka, was evaluated in mouse models of inflammation and sepsis by Stavropoulou et al. [48]. The potential modes of action associated with each type of Greek honey was compared to manuka honey as a standard. Researchers treated 8 female mice with a 30% solution of each of four types of honey (Arbutus, Chestnut, Fir, and manuka), as well as standard saline half an hour before injection with 1.5 mg/25 g body weight of LPS for the determination of cytokine concentrations of TNFα and IL-6 within mice serum samples. It was observed that supplementation of all three Grecian honeys to sepsis-induced mice significantly reduced TNFα-serum levels, as well as significantly reducing the expression of TNFα and iNOS from harvested liver tissue comparable to that of the manuka honey.

Another study using the Epirus and Crete varieties of fir honey, the LPS-suppression of the CYP1A1 gene, encoding the Phase 1 hepatic and extrahepatic enzymatic agent Cytochrome P450-1A1 involved in the metabolic activation of procarcinogens into reactive metabolites within hepatocytes, was reversed by honey [49]. Equal to this, the LPS-suppression of the hepatocyte levels of CYP2B10, the equivalent of CYP2B6 within humans, and chiefly responsible for the metabolism and detoxification of standard clinical drugs within hepatocytes, was also reversed by Evros chestnut and Epirus fir varieties of honeys [48]. The bacterial load in the harvested livers was reduced after administration of the Evros chestnut, Epiros fir, and Crete fir varieties of honey, and within harvested lungs reduced with administration of Epirus arbutus, Crete fir, and manuka honey samples [48]. 

**Figure 2:**
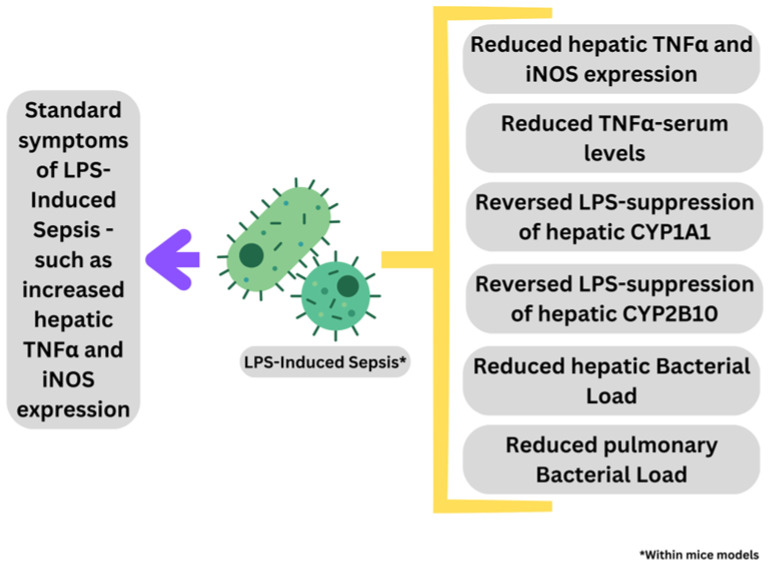
In vivo properties of Grecian honeys, with a manuka honey control, (yellow bracket on the right) application in LPS-Induced sepsis within mice–purple arrow (left) indicates extrapolated effects of LPS-Induced sepsis without Grecian honey application on mice (Figure is drawn by Authors).

Grecian honeys possessed unique anti-inflammatory and antibacterial properties–manifested through the reduction of mass bacterial translocation to distal tissues and systems within septic mice, as well as the significant reduction of pro-inflammatory mediators and markers involved within the septic process, with modes of action comparable to that of manuka honey [48,49]. Modes of action of Grecian honeys and manuka honey might include disruption of biofilm formation [50], morphological and structural modifications [51], depolarization of pathogen membranes, including integrity and potential blockage and disruption of efflux pump systems [52], and suppression of Quorum Sensing and reduction of autoinducer production [53]. A diagrammatic summary of the demonstrable in vivo properties of Grecian honey when applied to septic mice models, as discussed within the previous paragraphs, is presented in Figure 2.

Akankwasa assessed the antibacterial activity of undiluted African honey on the opportunistic pathogens *S. aureus* and *E. coli*, known pathogens of wound sepsis [54]. Through an agar well diffusion assay and a dye-reduction assay, zones of bacterial inhibition obtained were 10–15 mm for *E. coli*, and 14–15 mm for *S. aureus*, respectively, with a minimum inhibitory concentration range of 7.8125 mg/mL to 15.625 mg/mL, and a bactericidal inhibitory concentration of 125 mg/mL to 500 mg/mL—concluding that undiluted African honey demonstrates a distinct bacteriostatic and bactericidal property [54]. In another study Hussain also assessed the role of honey as a potential alternative treatment in the management of topical and systemic sepsis [55]. 

## 4. Viral Blood Infections 

### 4.1. Human Immunodeficiency Virus

Human Immunodeficiency Virus (HIV) is a commonly sexually transmitted viral infection of the CD4+ Helper-T Lymphocytes of the immune system [56]. Consequentially, CD4+ Helper-T Lymphocytes are colonized and destroyed in the process of viral replication, leading to the diminishing and weakening of the host’s immune system. Notably, such debilitating effects manifest as the host being unable to normally prevent infections from opportunistic pathogens, such as *Candida* spp., *Salmonella* spp., and more serious pathogens such as *Mycobacterium tuberculosis*, or even some cancers [56,57]. After prolonged infection with no means of treatment or therapeutic ablation of viral load, the infection will progress to Acquired Immunodeficiency Syndrome (AIDS), which is a chronic and life-threatening condition of a near-disabling of host’s immune system. The virus is commonly spread through sexual activity, but can also be spread through needle-sharing of narcotic paraphernalia, as well as perinatally from mother to child during gestation, childbirth, or through breast-feeding [58]. 

The symptoms of an HIV infection, as well AIDS, vary between patient and the stage of the infection the patient is within: acute HIV (primary infection) begins with flu-like symptoms 3–4 weeks after exposure, including other symptoms such as fever, muscle and joint pain, unintentional weight loss, and swollen lymph nodes, particularly of the neck, as well as a new and persistent cough, all of which last, potentially, for a few weeks before progressing [58]. Clinical latent HIV (chronic HIV) is usually asymptomatic, though the viral load is still present within the host’s system and immune cells—this stage of the infection may last years, especially if taking appropriate treatment and antiretrovirals [58]. Patients with clinical latent HIV may be described as asymptomatic. Symptomatic HIV can manifest as milder or more severe forms of the symptoms of acute HIV, but may include recurring bacterial pneumonia, oral thrush, or shingles [58]. If left untreated, within eight to ten years after exposure, the infection will progress to AIDS, and may manifest as chronic diarrhea, chronic fatigue, skin rash and irritation development, lesions, ulcers, and white spotting of the tongue and mouth, and recurring and persistent fever [58]. The HIV virus is categorized between HIV Type 1 (HIV-1) and HIV Type 2 (HIV-2), each with separate further subtypes, and it is recommended and accepted to give an immediate prescription of an antiretroviral regime after diagnosis of infection [59,60,61].

#### 4.1.1. Combinational Therapy for HIV by Nutraceutical Honey

Since the widespread awareness and research into the treatment of HIV infections, as well as AIDS and its associated comorbidities, across communities and within specific populations of people, novel agents of considerable efficacy have been developed and introduced into the standard regimes of many patients across the world [62]. Yusuf, et al. evaluated the implementation of Malaysian Taulang honey into the diet of asymptomatic HIV patients, with specific monitoring of potential viral load changes, CD4+ T-Helper Lymphocyte count changes, and any quality-of-life improvements [63]. Through a randomized controlled study, asymptomatic HIV patients (*n* = 95) with a CD4 T-Helper Lymphocyte count of 250–600 cell/mL and not on antiretrovirals were split within three separate groups of Taulang Honey Low (THL), Taulang Honey Intermediate (THI), and Taulang Honey High (THH), and given a 20 g dosage of honey for a six-month period. THL was given once daily; THI twice daily and THH thrice daily. Taulang honey showed potential as a adjuvant supplementation nutraceutical regime to improve quality-of-life and CD4+ Helper-T Lymphocyte count, as well as possible applications in reducing viral load, within asymptomatic HIV patients not receiving highly active antiretroviral therapy. Varieties of honey, including Taulang, manuka, Brazilian, and New Zealand Honeydew honey, contain essential amino acids as glutamic acid, aspartic acid, serine, tyrosine, glutamine, proline, tryptophan, and phenylalanine [64,65]. The study also hypothesized that, due to the presence of tryptophan and phenylalanine, two precursor components involved in the formation of the neurotransmitters serotonin and dopamine, within their sampled Taulang honey, this may be a reason for the observed improvements in psychological well-being [63]. 

### 4.2. SARS-CoV-2 (COVID-19 Virus)

The Severe Acute Respiratory Syndrome Coronavirus-2 (SARS-CoV-2) virus or COVID-19, is a positive-sense single-stranded RNA, enveloped, β-coronavirus of the Coronavirus family of viruses, and subgenus *Sarbecovirus*, and agent responsible for the widespread novel viral pneumonia that started the COVID-19 pandemic from 11 March 2020 to 5 May 2023 that killed 2.2 million people within Europe alone [66,67,68]. Symptoms of a COVID-19 infection are similar to that of a mild respiratory infection, namely involving a high fever, a new and persistent cough, nasal congestion, and chronic fatigue less than a week after exposure and can eventually progress to severe respiratory failure, and multiple organ failure [69].

Immunological studies in patients expressing severe COVID-19 symptoms, revealed lymphopenia—a clinically severe reduction of white blood cells in blood [70]—and an increased level of interleukin (IL)-6, IL-10, proinflammatory cytokines, as well as Granulocyte-Colony Stimulating Factor (G-CSF), Monocyte Chemoattractant Protein-1 (MCP-1), Tumor Necrosis Factor-α (TNFα), and Macrophage Inflammatory Protein-1α (MIP-1α) [69]. Within pulmonary tissue, the SARS-CoV viruses have been shown to produce the extensively understood Neutrophil and T-Lymphocyte chemoattractant IL-8 [71]. Through this production of IL-8, it is understood that this mass infiltration of Neutrophils and T-Lymphocytes, known inflammatory cells, are what induce pulmonary tissue damage via proinflammatory cytokine production and macrophagic production of Nitric Oxide [69].

#### 4.2.1. Combinational Therapy for SARS-CoV-2 by Nutraceutical Honey

Based on recent publications on the anti-inflammatory properties of stingless bee honey, Mustafa et al. hypothesized that a variety of honey, rich in the antioxidant phenolic compounds, phenolic acid, and flavonoids, as well as in polyphenols, could be implemented as a potential means to reduce the inflammatory pulmonary-based manifestations of COVID-19 [72]. This study was based on the research of Biluca et al. in 2019, where stingless bee honey (Meliponinae) demonstrated a reduction in TNFα by 23% and IL-6 secretion by 43.9%, significantly reducing the secretions of interferons, and inhibition of such interferons by 88.8% [73]. Stingless bee honey reduced the production of proinflammatory agents NOx, TNFα, IL-6, MCP-1, IL-12p70, INFγ, and IL-10 from the LPS-induced RAW264.7 macrophagic cell line [72,73]. This evaluation was concurrent with previous in-vivo analyses and assessments of the anti-inflammatory properties of Stingless Bee honey—demonstrating a capacity to decrease the circulation and concentration of other proinflammatory agents; namely, C-Reactive Protein (CRP), IL-1β, and IL-8 [72]. 

The study concluded emphasizing that viral manipulation of the normal cytological functions towards an exaggerated inflammatory response increases the severity and damage caused by the viral infection before replication. The heightened expression of cytokines like IL-6 can be regulated, potentially, with the correct study and application of stingless bee honey rich in bioactive polyphenolic content, which would exert a strong antioxidative and anti-inflammatory action on the proinflammatory cells and agents.

Abedi et al. evaluated the potential applications of honey and its constituent components as a possible treatment regime for a COVID-19 [74]. The anti-inflammatory properties of honey on proinflammatory cells and agents by the modulation and reduction of TNF, NF-kB pathways, PI3K/Akt, MAPK, T-Lymphocyte, B-Lymphocyte, RAS, and apoptosis signaling pathways could lead to a prevention of COVID-19 penetration and reproduction in host cells [74]. Additionally, Quercetin, a constituent of honey, has been shown to exert a protective property on host cells by inhibiting H^+^-ATPase of the lysosomal membrane of murine coronavirus specifically, preventing the shedding of viral coat after infiltration into cell [74]. This demonstration illustrates the modifying effect of honey on the lysosomal proteases involved in the penetration of the COVID-19 virus into host cells by the cleaving of viral surface proteins, and simultaneous contraction of the host and viral membranes—a strategy employed in the treatment of other retrovirus antiretrovirals such as that of HIV-positive patients. 

**Figure 3:**
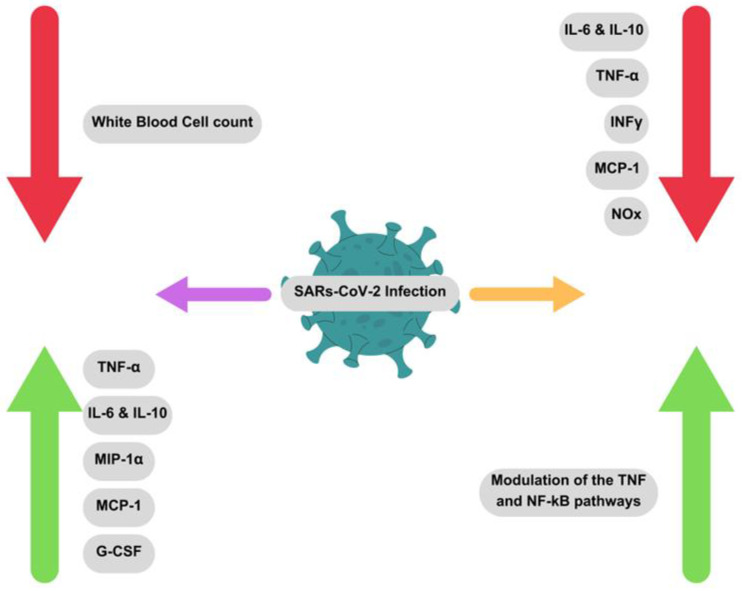
In vivo bioactive properties of stingless bee honey application during SARS-CoV-2 infections—purple arrow (left) indicates effects of SARS-CoV-2 without honey application upon the host, yellow arrow (right) indicates effects of honey application upon the host with a SARS-CoV-2 infection (Figure is drawn by Authors).

Principally, COVID-19 has shown an ability to encourage the manifestation of severe coagulopathy, increasing thrombotic complications and fibrotic activity [75]; hence, honey could reduce mediators of inflammation within lung infections. Kassim, et al. reported Gelam honey and its extracts inhibitory effects of Gelam honey and its extracts on nitric oxide and prostaglandin E2 in inflammatory tissues [76]. The studies concluded that honey reduced prostaglandin-E2, prostaglandin-2a, thromboxane-B2, it led to an increased concentration of nitric oxide end products, the agents involved in general vasodilation, and it increased the diffusion of oxygen into the blood and tissues in body [76]. A diagrammatic summary of the demonstrable in vivo properties of stingless bee honey when applied to an infection of SARS-CoV-2, is presented in Figure 3.

## 5. Parasitic Infections

Malaria is a dire and often life-threatening infection within humans that develops after being bitten by female mosquitos of the *Anopheles* genus that are infected with one of several species of insect protozoan parasites of the *Plasmodium* genus [77,78]. With current statistics available from the World Health Organization, the African region recorded 95% of the global cases of malaria in 2020, and 96% of the global deaths due to malaria. In 2021, it was believed that there were approximately 247 million live cases of malaria, with 619,00 approximate deaths due to the disease [78].

**Figure 4:**
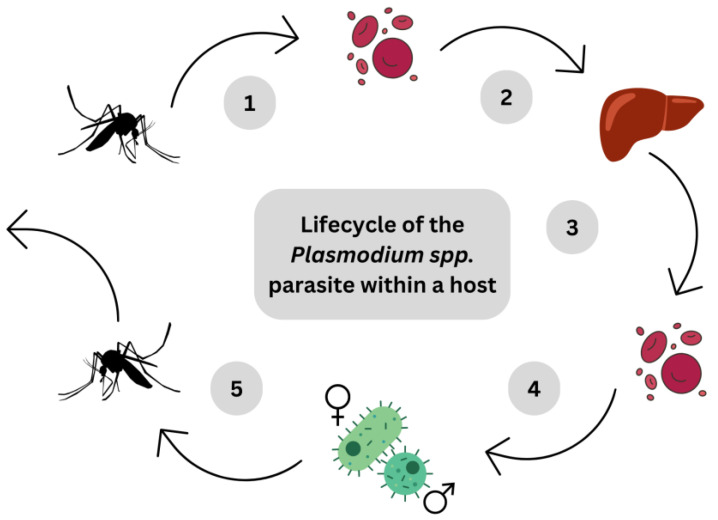
Simplified illustration of essential points within the lifecycle of the *Plasmodium* spp. parasite that is responsible for malaria, pertaining specifically to the stages within the host: (1) *Plasmodium* spp. sporozoites pass into the host bloodstream from bite of infected mosquito; (2) Sporozoites travel through host’s bloodstream to the host’s hepatocytes; (3) Sporozoites mature into schizonts and then merozoites within the hepatocytes, which are then released via cellular lysis; (4) Merozoites mature further mature into gametocytes within erythrocytes, and are released from the erythrocytes via cellular lysis; (5) Gametocytes, both male and female, are taken up and into an uninfected mosquito during a blood meal of an infected host (Figure is drawn by Authors).

The infection begins with the introduction of a *Plasmodium* spp. into the host bloodstream via an infected mosquito biting [79,80,81] with symptoms of fever, chills, nausea and vomiting, etc., and death if left untreated [82]. The manifestation of these symptoms correlated to the lysing of erythrocytes in blood, and the release of the toxin glycophosphatidylinositol—the putative malaria toxin. This acts on stimulating peripheral mononuclear cell release of both pro- and anti-inflammatory cytokines [79]. An illustration of the lifecycle of the *Plasmodium* spp. parasite can be seen in Figure 4.

### 5.1. Combinational Therapy for Malaria by Nutraceutical Honey

A preliminary evaluation of the potential antimalarial properties of combination extract of *Citrus aurantifolia* and honey derived from stingless bee honey (*Trigona* spp.), was studied on *Plasmodium*-*berghei*-infected mice. Therapy was compared to Artemisinin-based Combination Therapy (ACT) of Dihydroartemisinin-Piperaquine (DHP) chemotherapy [83]. Laksemi, et al. found the degree of parasite suppression with stingless bee honey, *Citrus aurantifolia*, and the product of their combination possessed a positive antimalarial property. However, the stingless bee honey demonstrated the higher degree of parasite suppression. 

The antimalarial potential of *Citrus aurantifolia* extract and stingless bee honey could be due to their phytochemical constituents, where the stingless bee honey contained flavonoids, tannins, phenols, alkaloids, and *Citrus aurantifolia* extract was rich in saponins, flavonoids, tannins, phenols, alkaloids, and steroids [83,84]. Flavonoids in stingless bee honey and lime extract have been suggested to exhibit some antimalarial properties, but more so these demonstrate a significant antimalarial activity due to synergistic interactions with artemisinin chemotherapy, although the exact and precise mechanisms of the action and interaction require further investigation for the conclusion of such roles [83,84,85].

## 6. Conclusions and Future Prospective

A variety of compounds have been analyzed in honey sourced from different geographical regions. A list of bioactivities including antioxidant, antimicrobial, anti-inflammatory, etc., give credence to the recommendation of honey as a natural therapeutic agent [11]. Studies have shown that honey could be used for the treatment of not only topical infections, but also for systemic infections. The bioactive molecule present in honey makes it a natural source possessing therapeutic properties for its fortification in food [86]. 

However, though numerous studies and reviews have been published, as of recently, for the promotion of honey as more than a naturally occurring edible substance with a potential as a novel nutraceutical, the material is not without its own inherent limitations—especially within clinical settings. Some of the main limitations that clinical application of honey’s inherent properties may face include: variability in its composition, highlighting that, although honey is composed of many beneficial and exploitable compounds, without strict, precise, and accurate standardization of different honey types, any therapeutic properties may vary between different geographical samples of honey or even samples of the same geographical type of honey harvested from different points in a particular season or the year; and processing methods, namely illustrating the various commercial processes that honey may pass through, such as filtration, pasteurization, etc., that may negatively impact the therapeutic properties of heat-sensitive compounds within honey, such as enzymes, phenols, peptides, and vitamins, and, thus, its therapeutic potential would be affected [62]. 

Clinical treatment with honey upon individuals who may suffer from any of the previously listed pathologies but are also diabetic or have any other condition which may require tracking of blood sugar levels may not be appropriate, as the primary constituent of all kinds of honey is sugar, namely glucose and fructose, and would spike blood sugar levels to degrees of concern for the individual and their health research; studies that conclude with positive results in line with their respective hypotheses may, unconsciously, be pushed for publication more frequently over equally valid studies presenting either negative or inconclusive findings, which then negatively impacts the body of literature and misjudges the capabilities of honey within clinical settings or as a novel therapy [62]. 

The previously mentioned limitations of honey are accepted and valid challenges that must be overcome and thoroughly thought through before final designation as a nutraceutical. This is, through itself, encouraging for its inclusion within more Randomized Clinical Trials pertinent to potentially new nutraceutical treatments for gastric infections and food discomforts [87]. Evidently, it was noticed in the review of published reports relating to the use of honey as an alternative treatment that honey appears to show some form of discrimination between pathogenic and non-pathogenic and unique aggregates of the normal gastrointestinal microflora [55]; novel antibiotics will ease the global concern of antimicrobial resistance for currently prescribed chemotherapeutic agents. This concept possesses necessity for further investigation, as well as providing new directions to study honey from different geographical sources, and their therapeutic mechanism against the activity of pathogens and pathogenic prevention could be exploited.

## Figures and Tables

**Table 1:** Bioactive properties and applications of manuka honey.

Activities and Applications of Honey	References
Antiproliferative capacity	[19]
Capture and reduction of free radicals	[17,18]
Ability to invade and evade detection and removal by bacterial efflux mechanisms	[22]
Synergistic capacity of MGO with other known antibacterial materials in destruction of Gram+ and Gram− bacteria	[23]
Estrogenic activity involving MCF-7 pro- and antiproliferation capacity, and cytotoxicity	[19,27,28]
Topical antibiofilm agent relevant to wound healing	[29]
Macrophagic stimulation in aid of tissue healing due to bacterial damage and infection	[30]
Pronounced antibacterial capacity	[31,32,33]
Topical treatment for burn wound healing	[34]
Promotion of healing post-craniomaxillofacial surgery, and reduction of complications associated with healing	[35]

## Data Availability

Not applicable.
